# Violence against healthcare workers in the middle of a global health crisis: what is it about policy and what to learn from international comparison?

**DOI:** 10.3389/fpubh.2023.1182328

**Published:** 2023-05-18

**Authors:** Ellen Kuhlmann, Michelle Falkenbach, Gabriela Lotta, Tim Tenbensel, Alexandra Dopfer-Jablonka

**Affiliations:** ^1^Department of Rheumatology and Immunology, Hannover Medical School, Hanover, Germany; ^2^Health and Health Systems, Faculty I, University of Siegen, Siegen, Germany; ^3^European Observatory on Health Systems and Policies, Brussels, Belgium; ^4^Department of Public Administration, Getulio Vargas Foundation, São Paulo, Brazil; ^5^Faculty of Medical and Health Sciences, Health Systems, University of Auckland, Auckland, New Zealand; ^6^German Center for Infection Research, Brunswick, Germany

**Keywords:** healthcare workforce, violence against healthcare workers, health policy, global health crisis, public health, COVID-19 pandemic, international comparison

## Abstract

**Introduction:**

Violence against healthcare workers is a global health problem threatening healthcare workforce retention and health system resilience in a fragile post-COVID ‘normalisation’ period. In this perspective article, we argue that violence against healthcare workers must be made a greater priority. Our novel contribution to the debate is a comparative health system and policy approach.

**Methods:**

We have chosen a most different systems comparative approach concerning the epidemiological, political, and geographic contexts. Brazil (under the Bolsonaro government) and the United Kingdom (under the Johnson government) serve as examples of countries that were strongly hit by the pandemic in epidemiological terms while also displaying policy failures. New Zealand and Germany represent the opposite. A rapid assessment was undertaken based on secondary sources and country expertise.

**Results:**

We found similar problems across countries. A global crisis makes healthcare workers vulnerable to violence. Furthermore, insufficient data and monitoring hamper effective prevention, and lack of attention may threaten women, the nursing profession, and migrant/minority groups the most. There were also relevant differences. No clear health system pattern can be identified. At the same time, professional associations and partly the media are strong policy actors against violence.

**Conclusion:**

In all countries, muchmore involvement from political leadership is needed. In addition, attention to the political dimension and all forms of violence are essential.

## Introduction

Violence against healthcare workers (HCWs) is a persistent and pressing concern, and the COVID-19 pandemic has added new threats. Systematic data and monitoring are still lacking, yet international organisations and mounting individual cases call to action, highlighting sharp increases and qualitatively new dimensions of hate, harassment, and severe violent attacks against HCWs (1–4). An increase in violence amidst a major global health crisis is exceptionally problematic, considering the dire need for HCWs who are subjected to immense pressures and run high risks of illness (5, 6). These attacks threaten individual HCWs and may even result in traumatisation and temporary absence due to illness. They also create long-term risks for the healthcare workforce (HCWF) and strain recruitment and retention efforts. Since women account for about 75% of HCWs in most countries, the gender-based and sexual violence dimensions, as well as the threats to nurses, are evident (1, 3, 6).

Increased violence against the HCWs comes at a critical point in the global health crisis when countries worldwide struggle to meet population health demands due to severe HCWF shortages (7–10). Given the resolute nature of the concern, its impact on health and care systems, and its detrimental effect on HCWs and gender equality, it is time that violence against HCWs is given much greater priority as a policy problem.

## Bringing health systems, policy and politics into the debate: using a country comparison to identify gaps

This perspective article brings policy and politics into the debate on violence against HCWs. Available evidence shows that violence was heightened during the pandemic, even in countries with formal democratic institutions and upper-middle to high-resourced healthcare systems. This raises questions as to whether and how institutional/systemic, epidemiological, and pandemic policy conditions shape the debate on violence. Applying a comparative lens and exploring the problem within various countries may help identify policy gaps and develop new policy solutions.

We have chosen a most different systems comparative approach concerning the epidemiological, political, and geographic contexts. In our research design (Table 1), Brazil (under the Bolsonaro government) and the United Kingdom (under the Johnson government) serve as examples of countries that were strongly hit by the pandemic in epidemiological terms while also displaying policy failures attributed to populist right-wing governments (12, 13). New Zealand and Germany represent the opposite. They serve as representatives of countries that managed the pandemic comparatively well under more moderate and balanced political constellations (14, 15). We refer to the period of the COVID-19 pandemic from its onset in 2020 until the end of 2022.

**Table 1:** Mapping the country sample: health system, workforce, and COVID-19 pandemic characteristics.

Item	Brazil	Germany	New Zealand	United Kingdom
Country profile				
Government/Leader	Jair Bolsonaro as President in a conservative extreme right-wing coalition, until December 2022.	Angela Merkel, coalition government led by Conservatives until October 2021; since then, Olaf Scholz, coalition of social democrats/ Green/ liberals.	Jacinda Ardern, Labour party-led coalition until October 2020, then single party majority.	Boris Johnson, Prime Minister for the conservative party until September 2022, prominent figure in the populist Brexit campaign/ anti-European Union (EU) membership referendum
Funding	Mainly by national taxes supplanted by some private insurance.	Mainly employer-employee contributions supplemented by little taxation and private contributions.	Mainly taxation supplemented by 14% out-of-pocket and 5% private insurance.	General taxation supplemented by National Insurance contributions (NICs).
Provision	Universal Health System (SUS), public, free and universal service provision, underfunded.	Social health insurance (SHI) system; well-resourced hospital and primary care sectors.	Hospitals publicly owned, primary care predominantly private, small business, 2010–18 decade of significant underfunding.	NHS system, massively underfunded.
Total health expenditure % GDP*	9.6	12.8	9.7	11.9
HCWF density* practising per 1,000				
• Physicians	2.15	4.53	3.53	3.18
• Nurses	1.55 (10.1)^#^	12.06	10.91	8.68
• Care personnel	n.a.	7.57	n.a.	18.47
COVID-19 epidemiology, cumulative deaths per million until February 2023	3,240.05	1,997.44	482.52	3,212.72
COVID-19 policy	Decentralised with denialism at the federal level; policies implemented locally by governors and majors.	Decentralised and multi-stakeholder based, with some centralised action.	Strongly centralised.	Strongly decentralised and multi-stakeholder based; limited political attention at the federal level, particularly during the first wave.
	Moderate lockdown/ local decisions.	Moderate to strong lockdown and social distancing policies; public funding to mitigate social effects.	Strong lockdowns nationally in 2020 and regionally in 2021.	Moderate to strong lockdown and social distancing policies; public funding to mitigate social effects.
	Lack of funding; vaccines applied only after pressure over the President.	Vaccines available and easy accessible.	Successful vaccination policy, except for inequitable rollout of vaccines.	Vaccines available and easy accessible.

Authors’ own table.

* OECD (11), data refer to 2021 or the latest available year.

# Methodological differences concerning nurses; Brazilian government data are much higher than OECD (11) data.

We rapidly assessed available data, policy responses and actors, and material on the discourse surrounding violence and actions taken against it in the four selected countries. A topic guide served as a framework for the comparative assessment, drawing on country expertise and secondary sources (media reports, documents, public data, and surveys).

### Making policy gaps visible

Our comparative assessment (Table 2) highlights two significant elements: the global dimension of violence against HCWs, and specific policy gaps that may hamper action taken to prevent violence. The results concerning the global dimension broadly reveal similar challenges in a country sample characterised by institutional and epidemiological differences in higher-middle and high-income countries (Table 1). This is an important finding because it suggests that violence occurs no matter how rich, developed or epidemiologically advanced a country may be. Therefore, increased funding and staffing are essential but insufficient to resolve the problem without additional measures. At the same time, we found some important differences related to policy and actors. Against this backdrop, a better understanding of policy gaps may pave the way for new opportunities for action both globally and in the national context.

**Table 2:** Comparative analysis of violence against healthcare workers, policy and actors.

Item	Cross-country comparative results
Data availability	
Accessible data	Generally limited data with only occasional studies and small surveys. The exception is the UK, where NHS staff surveys have been regularly conducted.
Monitoring availability for the COVID-19 period	There are no monitoring policies in place, and the evidence for trends during COVID-19 is poor. Nurses’ unions in New Zealand doubt the accuracy of official data. Germany has had police statistics available since 2022.
Policy and actors	
Public debate and media	President Bolsonaro supported attacks against HCWs during the pandemic in Brazil, while the media supported the HCWs. In the other countries, national and media support was geared toward the HCWs. In Germany, this support climaxed around the New Year’s Eve attacks on HCW, while New Zealand and the UK showed little increase in media attention during the pandemic.
Political radar	Political action can be found in the UK with the 2021 National Violence Prevention and Reduction Standard and the Spring 2022 campaign #WorkWithoutFear. In Germany, medical associations and the Länder have released some statements, and a centralised register system to monitor attacks was called for. In Brazil, unions and associations have called to action without government attention; in New Zealand, nurses have released statements.
Health policy, action and future plans	NHS England is working to establish a coherent approach for collecting data, with an aim to ensure alignment with the NHS Violence Prevention and Reduction Standard. In Germany, the policy is decentralised, and responsibility shifted to the organisational level; several hospitals and ambulances have increased security and support, and some pilot projects have been discussed. In New Zealand, responses are generally weak and decentralised, and nothing was on the agenda in Brazil.
Legal action	No specific action during COVID-19 for most countries aside from Germany, where attacks against HCWs have been registered separately in police statistics.
Professional associations	Professional associations are key actors in all countries, yet the relative contribution of doctors and nurses varies. During the pandemic, nurses seemed to be the strongest actors in Brazil and doctors in Germany, with New Zealand and the UK, taking a middle position. Paramedics also play a role.
Key actors engaged in the debate	The media and professional (nursing and/or medical) associations are the strongest actors in all countries. Paramedics, hospital organisations, and some institutional and government actors (Ministers of Health, Presidents/Chancellors) also play a role (centralised/NHS or decentralised/local).
The substance of the debate and action	
What groups of HCWs are addressed?	There is a focus on doctors, nurses, and paramedics, with some variation between countries; less attention to other groups. Germany reflects the professional hierarchy of medicine most strongly, while the NHS systems seem to be more inclusive, and Brazil prioritises nurses/carers.
Is gender-based and sexual violence addressed?	Usually not explicitly addressed; not systematically connected to an emergent sexual violence and harassment (#Metoo) debate in healthcare. Some signs of improved attention in Germany.
Is racialised violence addressed?	Usually not explicitly addressed, except in the UK, occasionally (mis)used by populist politics as a racialised anti-migration discourse in relation to the offenders, as observed in Germany.
Is the violence discourse connected to COVID-19?	Some connection in Germany and Brazil. Usually, no explicit connection in the UK and New Zealand, as violence was an issue pre-COVID, e.g., due to long waiting hours and underfunding. Some controversial evidence.
Is the political dimension addressed?	In Brazil, some connection to the populist radical right Bolsonaro government. In New Zealand and the UK, no explicit connections to the government but understaffing and underfunding have been major problems pre-COVID for years. In Germany, some connection to populist radical right movements surrounding anti-vaxxers and anti-abortion, and some connection to HCW shortages.

Authors’ own table, based on country case studies (Supplementary Tables S1–S4).

### The lack of data and monitoring hampers policy solutions

Available data is scattered, and access is generally limited in all countries. Evidence is mainly based on either criminal (police) statistics or surveys, both of which are limited in their ability to tell a holistic story. While pre-COVID survey data exists in New Zealand and the UK, suggesting that violence was a relevant health system problem before the pandemic, a lack of systematic data and monitoring systems makes it difficult to explore to what extent and why violence actually increased during the pandemic. Insufficient empirical evidence hampers a critical debate and the development of effective policy solutions and also opens the door for various forms of interest-driven politics.

### Policy and actors: more involvement from political leadership is needed

Strong political leadership and effective policies play a critical role in aiding HCWs. Unfortunately, political leadership in the examined countries has remained sparse; however, health professional associations (doctors, nurses, and paramedics) have proven to be important and valuable supporters. The nurses’ associations appear to play the biggest supportive role in Brazil, while doctors’ associations take the lead in Germany. The associations in New Zealand and the UK also matter, including hospital organisations and paramedics associations.

The policy initiatives among the cases reflect country-specific governance arrangements, particularly centralised vs. decentralised governance structures. The most centralised efforts can be seen in the UK, where the NHS is working to improve data collection and analysis across NHS trusts, propelled by the #WorkWithoutFear campaign. The associations and some regional (Länder) governments called for a centralised register system to monitor attacks in Germany. In addition, legal action was taken to improve policy statistics; here, we can observe more decisive action taken on the organisational and operational levels of governance (e.g., increasing security services and technical support). The other two countries showed limited initiative. Overall, sensitivity to the problem seems to be increasing, yet change is incremental, action is limited to piecemeal work, and actor collaboration is poorly developed (reflecting professional silos). Much more involvement from political leadership is necessary to set the agenda throughout government and society, thereby increasing the likelihood of action and, hopefully, changing the status quo on violence against the HCW.

### Substance: gender-blind and insufficent attention to the political dimension

If violence is addressed, this mainly relates to doctors and nurses as the most significant groups, with some country-specific variation. However, health workforce policy primarily focuses on health labour markets and system needs rather than on HCWs as human beings with specific conditions and needs related to age, sex, gender, ethnicity/race, and other social positions. Ignoring the human behind every HCW seriously obstructs the opportunity to protect HCWs better and improve prevention. This creates additional policy gaps exacerbating existing social inequalities in the HCWF, especially in professional groups with more women and migrant HCWs.

The connection to the COVID-19 pandemic was substantial, especially in Germany and Brazil, where increased violence against HCWs was most prominent. New Zealand and especially the United Kingdom have faced the challenge of violence well before the pandemic; however, only the latter country has developed the beginnings of a strategy to combat it. We generally observed an overall lack of attention to the political dimension of violence against HCWs. However, there were also some examples of explicit connections to the populist radical right movement in Brazil.

## Conclusion

Violence against HCWs is and will remain a problem long after the pandemic subsides. If political action is not taken, HCWs will have an additional reason to leave their profession and workplace, and potential candidates will be made to consider the increasing risks of HCWs and pursue a different line of work. In a time when countries across the globe are struggling with HCW retention and recruitment protecting the health and care workforce is essential. Getting support and protection right enhances the retention of the existing workforce and will attract new generations of HCWs. Improved working conditions, mental health, and physical safety of HCWs are an obligation not only of organisations and employers but also of governments and policymakers. This will require governments to prioritise developing feasible and effective policy responses that tackle the many individual risk factors the HCWs face on a daily basis, as well as the health workforce and system-related risks.

## Data availability statement

The original contributions presented in the study are included in the article/Supplementary material, further inquiries can be directed to the corresponding author.

## Author contributions

EK and MF had the idea, developed the framework, supervised the analysis, and prepared a draft. EK, MF, GL, TT, and AD-J collected the country cases, contributed to the analysis, commented on the draft, and have read and approved the final version. All authors contributed to the article and approved the submitted version.

## Conflict of interest

The authors declare that the research was conducted in the absence of any commercial or financial relationships that could be construed as a potential conflict of interest.

## Publisher’s note

All claims expressed in this article are solely those of the authors and do not necessarily represent those of their affiliated organizations, or those of the publisher, the editors and the reviewers. Any product that may be evaluated in this article, or claim that may be made by its manufacturer, is not guaranteed or endorsed by the publisher.
