# Supplementary material for: Violence against healthcare workers in the middle of a global health crisis: what is it about policy and what to learn from international comparison?
Source: Front Public Health. 2023 May 18;11:1182328. doi: 10.3389/fpubh.2023.1182328 (PMC10232894; doi:10.3389/fpubh.2023.1182328)
Supplement: Supplementary file 1 [file Table_1.DOCX]

**Supplemental material**

Table S1. Country case Brazil

| **Item** | **Brazil** |
| --- | --- |
| Country profile |  |
| Government/leader | - Jair Bolsonaro as president in a conservative extreme right-wing coalition |
| Funding | - Mainly by national taxes supplanted by some private insurance |
| Provision | - Universal Health System (SUS), public, free and universal service provision, underfunded |
| Total health expenditure | - % GDP*: 9.6 |
| HCWF density* practicing per 1000 | - Physicians: 2.15; Nurses: 1.55 (10.1)^#;;^ Care personnel: n.a. |
| COVID-19 epidemiology | - Cumulative deaths per million until February 2023: 3,240.05 |
| COVID-19 policy | - Decentralised with denialism at the federal level; policies implemented locally by governors and majors. - Moderate lockdown/ local decisions. - Lack of funding; vaccines applied only after pressure over the President. |
| Data availability | |
| Accessible data | - Few studies from associations and HCW unions, occasionally published. - Data overall very poor. |
| Monitoring availability for COVID-19 pandemic period | - No monitoring policies and national data. - Occasionally data and research are collected by unions and association, with a focus on nurses. - Poor evidence of trends during COVID-19. |
| Policy and actors | |
| Public debate and media | - The president supported attacks against HCWs during the Covid-19 pandemic, with some public statements. - The media criticised the President and supported HCWs. - Media attention to cases of violence, mainly in 2020. |
| Political radar | - No national police reporting. - Unions and associations called to action, but no government attention. - A specific event got strong media attention in support of nurses: a Bolsonaro supporter acted violently against nurses who demonstrated for support in front of the Congress; the President did not condemned the attack. |
| Health policy, action and future plans | - Not on the agenda of the Bolsonaro government. - Some efforts from unions and professional associations. |
| Legal action | - No specific action. - Violence against HCWs is legally considered as any other kind of violence and follows the same procedures. |
| Professional associations | - Nurses develop monitoring and campaigns, and launch media reports. - No specific action taken by doctors. |
| Key actors engaged in the debate | - Unions and professional associations and the media. |
| Substance of the debate and action | |
| What groups of HCWs are addressed? | - No government action. - Media highlights attacks mainly against nurses, after an attack in front of the congress, but also doctors are also mentioned. |
| Is it connected to COVID-19? | - Bolsonaro stimulated violence against HCWs as he denied the COVID crisis, supported the use of non-scientific proved medicine, and criticised HCWs engaged in pandemic protection. - Some situations of violence were reported by the national media. |
| Is gender-based and sexual violence addressed? | - Usually no. |
| Is racialised violence addressed? | - Usually no. |
| Is the political dimension addressed? | - Usually no. - The political sphere stimulated violence through public speeches denying the pandemic, and criticising HCWs who were fighting the pandemic and/or did not agree to distribute medicine with no scientific evidence (e.g. Chloroquine). |

Source: authors’ own table

***** OECD, 2023; data refer to 2021 or latest available year

^#^ Methodological differences concerning nurses; Brazilian government data are much higher than OECD data

**References**

Almeida NR, Bezerra Filho JG, Marques LA. Analysis of the scientific production on violence at work in hospital services. *Rev Bras Med Trab.* (2017) 15(1):101–12.

Alves JC dos S, Carvalho AR, Santos CFN dos, Gomes TMC, Santos TR dos, Santos VM, et al. Violência contra os profissionais de saúde no contexto atual brasileiro: Uma abordagem histórica / Violence against health professionals in the current Brazilian context: A historical approach. *Brazilian Journal of Developmen.* (2022) 8(1):3699–725. <https://doi.org/10.34117/bjdv8n1-243>

Barros C, Sani A, Meneses RF. Violência contra profissionais de saúde: Dos discursos às práticas. *Configurações. Revista Ciências Sociais.* (2022) 30:33–46.

Lotta G, Fernandez M, Correa M. The vulnerabilities of the Brazilian health workforce during health emergencies: analysing personal feelings, access to resources and work dynamics during the COVID-19 pandemic. *Int J Health Plann Mgnt.* (2021) 36(S1):42–57.

Magri G, Fernandez M, Lotta G. Inequalities in the middle of a crisis: an analysis of health workers during the COVID-19 pandemic from the profession, race and gender perspectives. *Cien Saude Cole.* (2022) 27:4131–44. doi: 10.1590/1413-812320222711.01992022

Massard da Fonseca E, Nattrass N, Bolaffi Arantes L, Bastos FI. COVID-19 in Brazil. In Greer SL, King EJ, Massard da Fonseca E, Peralta-Santos A, editors. Coronavirus Politics. The Comparative Politics and Policy of COVID-19. Michigan: University of Michigan Press, 2021: 494–510. Available online at: <https://www.jstor.org/stable/10.3998/mpub.11927713.29> (accessed 28 February 2023)

OECD.stat. Health workforce. Paris: OECD, 2023. Available online at: <https://www.oecd.org/els/health-systems/health-data.htm> (accessed 28 February 2023)

Table S2. Country case Germany

| **Item** | **Germany** |
| --- | --- |
| Country profile | |
| Government/  leader | - Angela Merkel, coalition government led by Conservatives until October 2021; since then, Olaf Scholz, coalition of social democrats/ Green/ liberals. |
| Funding | - Mainly employer-employee contributions, supplemented by little taxation and private contributions |
| Provision | - Social health insurance (SHI) system; well-resourced hospital and primary care sectors |
| Total health expenditure * | - % GDP: 12.8* |
| HCWF density* practicing per 1000 | - Physicians: 4.53; Nurses: 12.06; Care personnel: 7.57 |
| COVID-19 epidemiology | - Cumulative deaths per million until February 2023: 1,997.44 |
| COVID-19 policy | - Decentralised and multi-stakeholder based, with some centralised action. - Moderate to strong lockdown and social distancing policies. - Public funding to mitigate social effects; vaccines available and easy accessible. |
| Data availability | |
| Accessible data | - Few surveys from associations. Police statistics occasionally published. Data are poor. |
| Monitoring availability for COVID-19 pandemic period | - No monitoring policies but action through revision of policy statistics. - Police statistics available; since 1 January 2022 they include attacks against HCWs reported to the policy; attacks may also be registered in a specific category ‘politically motivated’ criminal offenses (e.g. those related the radical right. - Poor evidence of trends during COVID-19. |
| Policy and actors | |
| Public debate and media | - Media attention, some reports, some social media action. - Recent government attention after the New Year’s Eve attacks on HCWs; the current Chancellor condemned the attacks; the previous Minister of Health called for legal action. - Immediately after the New Year’s Eve attacks, some connection to racialised populist migration policy concerning the offenders but do continuing trend. |
| Political radar | - Medical Profession General Assembly May 2022 called on Länder (regional) governments to establish centralised register systems to monitor attacks against emergency and medical staff. - Some statements by the medical profession and hospital society. - Some statements by the Länder governments that are responsible for hospitals and some other services. - Few activities by the nursing associations, but calls on employers to improve occupational safety. |
| Health policy, action and future plans | - Some plans to improve protection of HCWs. - Some plans to protect HCWs providing abortion services. - Trade associations improved reporting and called on government to take action. - Many hospitals, some emergency care services, and office-based physicians scaled-up or introduced private security services. - Health policy shifts responsibility to the hospital level. - Hospital pilot projects, e.g. training HCWs with the policy, establishing silent alarms. |
| Legal action | - Since 1 January 2022, attacks against staff members, organisations and cars belonging to the healthcare system are registered separately in the police statistic. |
| Professional associations | - The medical profession addresses the problem and strongly calls on governments to take action. - Little efforts by the nursing profession and the Unions. |
| Actors engaged in the violence debate | - Medical profession and the media are major actors. - Some action by the hospital society, paramedics, and emergency services. |
| Substance of the debate and action | |
| What groups of HCWs are addressed? | - All groups, but particular focus on emergency and frontline HCWs including GPs and parademics, sometimes connected to vaccination provision. - Media debate is mainly focused on doctors, emergency staff and paramedics but some reports on nurses. |
| Is violence connected to COVID-19? | - Some linkage to lockdown policies and social distancing measures. - Some connection to vaccination provision. |
| Is gender-based and sexual violence addressed? | - Usually no. - Sensitivity has improved but the discourse is rarely connected to violence against HCWs. - Some connection to the attacks against abortion services. - Few reports mention sexual violence against nurses. |
| Is racialised violence addressed? | - Mostly no. - A racialised discourse emerged concerning the offenders, which is linked to the refugee debate and primarily targeting young Arab/ Muslim men. |
| Is the political dimension addressed? | - Occasionally connected to radical right-wing movements and antivaxxers. - Some connection to the radical anti-abortion movement. - Some connection to HCW shortages and high stress and workload during COVID. |

Source: authors’ own table

***** OECD, 2023; data refer to 2021 or latest available year

^#^ Methodological differences concerning nurses

**References**

Aerztebaltt.de. 276 Angriffe auf Mitarbeiter des Gesundheitswesens. 3 October 2022. Available online at: <https://www.aerzteblatt.de/nachrichten/archiv?jahr=2022&monat=10&tag=3> (accessed 27 February 2023)

Aerzteblatt.de. Gewalt gegen Gesundheitspersonal: Ruf nach schärferem Vorgehen. 10 October 2022. Available online at: <https://www.aerzteblatt.de/nachrichten/137984/Gewalt-gegen-Gesundheitspersonal-Ruf-nach-schaerferem-Vorgehen> (accessed 27 February 2023)

Aerzteblatt.de. Münster, ÄKWL, Gewalt gegen Rettungskräfte konsequent bestrafen, 23. January 2023. Available online at: <https://www.aerzteblatt.de/nachrichten/140603/Gewalt-gegen-Aerzte-und-Rettungskraefte-konsequent-bestrafen> (accessed 27 February 2023)

ARD Politikmagazin, Report Mainz und Zeit online. Gewalt gegen Pflegende, 19 September 2021. Available online at: <https://www.ardmediathek.de/video/report-mainz/gewalt-gegen-pflegende/das-erste/Y3JpZDovL3N3ci5kZS9hZXgvbzE1MzA3ODE>; see also, <https://m.facebook.com/DasErste/videos/162275369322922/> (accessed 27 February 2023)

Blümel M, Spranger A, Achstetter K, Maresso A, Busse R. Germany: Health System Review. *Health Syst Transit* (HiT) 2020;22(6):1–272. Available online at: <https://apps.who.int/iris/bitstream/handle/10665/341674/HiT-22-6-2020-eng.pdf> (accessed 27 February 2023)

Dienstzimmer.com. Gewalt gegen Pflegekräfte, 2021. Available online at: <https://dienstzimmer.com/gewalt-gegenueber-pflegekraeften/> (accessed 27 February 2023)

FK. Gewalt gegen Pflegekräfte. *Pflegezeitschrift.* (2018) 71:60. <https://doi.org/10.1007/s41906-018-0600-z>

OECD.stat. Health workforce. Paris: OECD, 2023. Available online at: <https://www.oecd.org/els/health-systems/health-data.htm> (accessed 22 January 2023)

Pflegekammer RLP. Gewalt gegen Pflegekräfte, no year. Available online at: <file:///C:/Users/asus/Downloads/PoPa_Gewalt_final%20(3).pdf> (accessed 22 January 2023)

Pflegen-online.de. Mehr als jede zweite Pflegekraft 2020/21 sexuell belästigt, 18 August 2021. Available online at: <https://www.pflegen-online.de/mehr-als-jede-2-pflegekraft-202021-sexuell-belaestigt> (accessed 22 January 2023)

Rechtsdepesche Gesundheitswese. Gegen Gewaltsituationen: Polizistin trainiert Pflegekräfte. 21 September 2021. Available online at: <https://www.rechtsdepesche.de/polizistin-trainiert-pflege-gewalt/> (accessed 22 January 2023)

Rechtsdepesche Gesundheitswesen. Gewalt: Übergriffe gegen Pflegekräfte nehmen zu. 16 December 2021. Available online at: <https://www.rechtsdepesche.de/gewalt-gegen-pflegekraefte/> (accessed 22 January 2023)

Schablon A, Wendeler D, Kozac A, Nienhaus A, Steinke S. Belastungen durch Aggression und Gewalt gegenüber Beschäftigten der Pflege- und Betreuungsbranche in Deutschland – ein Survey. Hamburg: Berufsgenossenschaft für Gesundheitsdienst und Wohlfahrtspflege (BGW), 2018. Available online at: <https://www.bgw-online.de/resource/blob/22246/3cb1bd64d7709df9c977f5c0b2c14121/studie-gewalt-mitteilungen-data.pdf> (accessed 22 January 2023)

Schuffenhauer H, Güzel-Freudenstein G. Gewalt gegen Pflegende in Notaufnahmen. *ASU Zeitschrift für medizinische Prävention.* (2019) 6:386–393. Available online at: <https://www.asu-arbeitsmedizin.com/wissenschaft/gewalt-gegen-pflegende-notaufnahmen> (accessed 28 February 2023)

Schuffenhauer H. Gewalt gegen Pflegende in Notaufnahmen. *ASU Arbeitsmed Sozialmed Umweltmed.* (2022) 57:98–105. doi:10.17147/asu-1-167090

Table S3. Country case New Zealand

| **Item** | **New Zealand** |
| --- | --- |
| Country profile | |
| Government/leader | - Jacinda Ardern, Labour party-led coalition until October 2020, then single party majority. |
| Funding | - Mainly taxation supplemented by 14% out of pocket and 5% private insurance. |
| Provision | - Hospitals publicly owned, primary care predominantly private, small business, 2010-18 decade of significant underfunding. |
| Total health expenditure | - % GDP*: 9.7 |
| HCWF density* practicing per 1000 | - Physicians: 3.53; Nurses: 10.91; Care personnel: n.a. |
| COVID-19 epidemiology | - Cumulative deaths per million until February 2023: 482.52 |
| COVID-19 policy | - Strongly centralized. - Strong lockdowns nationally in 2020 and regionally in 2021. - Successful vaccination policy, except for inequitable rollout of vaccines. |
| Data availability | |
| Accessible data | - Only pre-COVID nationwide data. - Some data obtained from occasional Official Information Act requests. - Some local data from Canterbury and West Coast. |
| Monitoring availability for COVID-19 pandemic period | - No monitoring procedures for reporting data. - Some data provided in response to Official Information Act requests from Nurses Union and media outlets. - Poor evidence of trends during COVID; Nurses Union doubts accuracy of official data. |
| Policy and actors | |
| Public debate and media | - Numerous reports, mostly local news outlets. - Media reports mostly pre-COVID or related to data collected prior to 2020. - Worbksafe report. |
| Political radar | - New Zealand Nurses Organisation report. |
| Health policy, action and future plans | - Decentralised responses, District Health Board responsibility – prior to their centralisation in July 2022. |
| Legal action | - No specific action. |
| Professional associations | - New Zealand Nurses Organisation is active in raising the issue. - New Zealand Medical Association and Resident Doctors Association. |
| Key actors engaged in the debate? | - Nurses organisations, junior doctors union, public service union, District Health Boards. |
| The substance of the debate and action | |
| What groups of HCWs are addressed? | - Nurses. - Emergency department staff. - Mental health units. |
| Is it connected to COVID-19? | - Some media linkage of increased violence to COVID restrictions. - Linkages mainly noted in 2022, during Omicron wave, and attributed to public fatigue and frustration with COVID restrictions, e.g. to hospital visitors. |
| Is gender-based and sexual violence addressed? | - Not explicitly. - Some reports mention sexual violence against nurses. |
| Is racialised violence addressed? | - Not defined as an aspect of the problem. |
| Is the political dimension addressed? | - No, but occasionally pre-COVID structural deficits of the healthcare system were mentioned, like underfunding and understaffing |

Source: authors’ own table

***** OECD, 2023; data refer to 2021 or latest available year

**References**

Bromfield N, McConnell A. Two routes to precarious success: Australia, New Zealand, COVID-19 and the politics of crisis governance. *Int Rev Adm Sci.* (2021) 87(3):518–35. <https://doi.org/10.1177/0020852320972465>

Canterbury District Health Board. RE Official Information Act request CDHB 10774 and WCDHB 9641. Christchurch, 2 February 2022. Available online at: <https://www.cdhb.health.nz/wp-content/uploads/b2566418-cdhb-10774-wcdhb-9641-violence-healthcare-staff-nurses.pdf> (accessed 28 February 2023)

Local democracy reporting. Covid-19: Union and frontline worker say staff at Middlemore Hospital facing increasing abuse. 8 March 2022. Available online at: <https://www.stuff.co.nz/national/politics/local-democracy-reporting/300534812/covid19-union-and-frontline-worker-say-staff-at-middlemore-hospital-facing-increasing-abuse> (accessed 28 February 2023)

New Zealand Work Research Institute. Maori care and support workers: Data from the 2019 New Zealand Care Workforce Survey. 2022. Available online at: <https://workresearch.aut.ac.nz/__data/assets/pdf_file/0003/675084/Maori-Care-and-Support-Workers-Report-2022.pdf> (accessed 28 February 2023)

News. Abuse of ED staff widespread in NZ hospitals, says doctors. May 2022. Available online at: <https://www.1news.co.nz/2022/05/06/abuse-of-ed-staff-widespread-in-nz-hospitals-says-doctor/> (see also video link) (accessed 28 February 2023)

New Zealand Nurses Organisation (NZNO). Position Statement. Auckland: NZNO, no date. Available online at: <https://www.nzno.org.nz/LinkClick.aspx?fileticket=JpI7UyOnRCw%3D&portalid=0> (accessed 28 February 2023)

Nz.herald.co.nz. Threats, slaps, kicking: Report highlights extent of violence against health workers. 12 April 2022. Available online at: <https://www.nzherald.co.nz/nz/threats-slaps-kicking-report-highlights-extent-of-violence-against-health-workers/W7C37RWP2SOZB5N6C6T4E7CWNM/> (accessed 28 February 2023)

New Zealand Medical Association (NZMA). NZ Healthcare organisations condemn violence against healthcare workers. Auckland: NZMA, no date. Available online at: <https://journal.nzma.org.nz/media-releases/nz-healthcare-organisations-condemn-violence-against-healthcare-workers> (accessed 28 February 2023)

OECD.stat. Health workforce. Paris: OECD, 2023. Available online at: <https://www.oecd.org/els/health-systems/health-data.htm> (accessed 28 February 2023)

Te Whatu Ora New Zealand. Violence against healthcare staff. Canterbury: Health New Zealand, 2022. Available online at: <https://www.cdhb.health.nz/about-us/document-library/cdhb-10774-wcdhb-9641-violence-healthcare-staff-nurses/> (accessed 28 February 2023)

Table S4. Country case United Kingdom

| **Item** | **United Kingdom** |
| --- | --- |
| Country profile | |
| Government/leader | - Boris Johnson, Prime Minister for the conservative party from 2019-2022, prominent figure in the successful Vote Leave campaign for Brexit in the 2016 European Union. (EU) membership referendum. |
| Funding | - General taxation supplemented by National Insurance contributions (NICs). |
| Provision | - NHS system, massively underfunded. |
| Total health expenditure | - % GDP*: 11.9 |
| HCWF density* practicing per 1000 | - Physicians: 3.18; Nurses: 8.68; Care personnel: 18.47 |
| COVID-19 epidemiology | - Cumulative deaths per million until February 2023: 3,212.72 |
| COVID-19 policy | - Strongly decentralised and multi-stakeholder based. - Moderate to strong lockdown and social distancing policies; public funding to mitigate social effects; vaccines available and easy accessible. |
| Data availability | |
| Accessible data | - Some studies; a 2021 NHS staff survey of almost 600,000 responses from 220 NHS trusts. - Government plans for violence and abuse data from across the NHS to be reported nationally based on 2018 Assaults on Emergency Workers (Offences) Act (Department of Health and Social Care. |
| Monitoring availability for COVID-19 pandemic period | - No monitoring policies. - Pre-COVID data available; national data collection on NHS staff disbanded in 2016; but data available on the ambulance sector. - Poor evidence of trends during COVID-19; YouGov 2022 reported significant rise in recent years. |
| Policy and actors | |
| Public debate and media | - NHS staff survey 2021 does not report evidence of an increase in attacks. - Media reports mostly pre-COVID. - 2022 media reports suggest that health professionals, specifically paramedics are being attacked every hour due to slow response times. - Assaults on NHS staff in Scotland have increased by 34% since 2019. |
| Political radar | - 2021 [National Violence Prevention and Reduction Standard](https://www.england.nhs.uk/publication/violence-prevention-and-reduction-standard/). - Spring 2022 campaign #WorkWithoutFear. |
| Health policy, action and future plans | - NHS England are working with NHS trusts to establish a consistent, coherent approach to inform evidence-based solutions for collecting data on violence, with an aim to improve data collection across all trusts and analyse data fields to ensure alignments to the NHS Violence Prevention and Reduction Standard. - Open source, desk-top exercise to explore what data, evidence and research are available; focus on incidents against NHS staff, causation, themes and trends, and sectors and staff groups most at risk. - People Plan for 2020/21 to explore ways to protect NHS workers and deliver a safe and inclusive working environment. |
| Legal action | - No specific action during COVID-19, but pre-COVID. - 2018 zero-tolerance approach aims to protect the NHS workforce against deliberate violence and aggression from patients, their families and the public, and to ensure offenders are punished quickly and effectively. - Assaults on Emergency Workers (Offences) Act 2018. |
| Professional associations | - NHS. - British Medical Association. |
| Key actors engaged in the debate? | - NHS, doctors, nurses associations, hospitals, paramedics, the media. |
| Substance of the debate and action | |
| What groups of HCWs are addressed? | - All groups, but particular focus on doctors, nurses, and paramedics. |
| Is it violence discourse connected to COVID-19? | - Usually no explicit connection as violence was an issue pre-COVID, e.g. due to long waiting hours and underfunding. - Evidence that attacks are still present during COVID, yet controversial evidence concerning an increase. |
| Is gender-based and sexual violence addressed? | - Not explicitly addressed. |
| Is racialised violence addressed? | - Ethnicity was the most common characteristic associated with harassment and discrimination from patients in primary care. |
| Is the political dimension addressed? | - No explicit connection to the Johnson government and/or Brexit, as understaffing and underfunding have been major problems of the NHS pre-COVID since years. |

Source: authors’ own table

***** OECD, 2023; data refer to 2021 or latest available year

^#^ Methodological differences concerning nurses

**References**

Andrew G. Criminal acts of violence at UK GP surgeries almost double in five years. The Guardian, 2022. Available online at: <https://www.theguardian.com/society/2022/may/31/criminal-acts-of-violence-at-uk-gp-surgeries-almost-double-in-five-years> (accessed 28 February 2023)

Campbell D. NHS staff face rising tide of abuse from patients provoked by long waits. The Guardian, 2021. Available online at: <https://www.theguardian.com/uk-news/2021/oct/10/nhs-staff-face-rising-tide-of-abuse-from-patients-provoked-by-long-waits> (accessed 28 February 2023)

Campbell D. Violence against NHS staff in England reaches five-year high. The Guardian, 2018. Available online at: <https://www.theguardian.com/society/2018/oct/31/violence-against-nhs-staff-in-england-reaches-five-year-high> (accessed 28 February 2023)

Davidson L. Violence against nurses could see body cameras rolled out across the NHS. The Sun, 2018. Available online at: <https://www.thesun.co.uk/news/7623969/violence-against-nurses-body-cameras/> (accessed 28 February 2023)

Department of Health and Social Care. Stronger protection from violence for NHS staff. London: NHS, 2018. Available online at: <https://www.gov.uk/government/news/stronger-protection-from-violence-for-nhs-staff>(accessed 28 February 2023)

Kirk I. Three in 10 healthcare workers say they experience violence from patients at least once a year. YouGov, 2022. Available online at: <https://yougov.co.uk/topics/health/articles-reports/2022/02/01/three-10-healthcare-workers-say-they-experience-vi> (accessed 28 February 2023)

National Health Service (NHS) Health Education England. Experiences of Racial Discrimination and Harassment in London Primary Care. London: NHS, 2022. Available online at: <https://www.hee.nhs.uk/sites/default/files/documents/Pan-LondonDiscrimination%26RacismPrimaryCareSurvey_Final.pdf> (accessed 28 February 2023)

National Health Service (NHS) 75 England*. Violence prevention and safety.* London: NHS, 2022. Available online at: <https://www.england.nhs.uk/supporting-our-nhs-people/health-and-wellbeing-programmes/violence-prevention-and-safety/> (accessed 28 February 2023)

National Health Service (NHS). We are the NHS: People Plan 2020/21 action for us all. London: NHS, 2020. Available online at: [www.england.nhs.uk/ournhspeople](http://www.england.nhs.uk/ournhspeople) (accessed 28 February 2023)

O’Dowd A. Third of London’s primary care workforce say they have faced racial abuse at work. *BMJ.* (2022) 377:o1171. <https://doi.org/10.1136/BMJ.O1171>

OECD.stat. Health workforce. Paris: OECD, 2023. Available online at: <https://www.oecd.org/els/health-systems/health-data.htm> (accessed 28 February 2023)

Roborgh S, Fast L. Healthcare workers are still coming under attack during the coronavirus pandemic. Manchester: Manchester University, 2020. Available online at: <https://www.manchester.ac.uk/discover/news/healthcare-workers-are-still-coming-under-attack-during-the-coronavirus-pandemic/> (accessed 28 February 2023)

Social Partnership Forum. (2020). Violence prevention and reduction standard. London: NHS, 2020. Available online at: <https://www.england.nhs.uk/publication/violence-prevention-and-reduction-standard/> (accessed 28 February 2023)

UK Parliament. Assaults on Emergency Workers (Offences) Act 2018. London: UK Parliament, 2018. Available online at: <https://bills.parliament.uk/bills/2058> (accessed 28 February 2023)

Williams GA, Rajan S, Cylus JD. COVID-19 in the United Kingdom: how austerity and a loss of state capacity undermined the crisis response. In Greer SL, King EJ, Massard da Fonseca E, Peralta-Santos A, editors. Coronavirus Politics. The Comparative Politics and Policy of COVID-19. Michigan: University of Michigan Press, 2021: 215–34. DOI:[10.3998/mpub.11927713](https://doi.org/10.3998/mpub.11927713)

Williams M. “Shocking”: Violent assaults on Scots NHS staff rise by over a third since before the pandemic. HeraldScotland, 2022. Available online at: <https://www.heraldscotland.com/news/homenews/23102704.shocking-violent-assaults-scots-nhs-staff-rise-third-since-pandemic/> (accessed 28 February 2023)
